# Characteristics of SLS-made 3D gyroid cubic lattice nanoporous polyamide membrane

**DOI:** 10.1371/journal.pone.0324326

**Published:** 2025-06-09

**Authors:** Saleh Ahmed Aldahash, Mohammad Kashif Uddin

**Affiliations:** 1 Department of Mechanical and Industrial Engineering, College of Engineering, Majmaah University, Al-Majmaah, Saudi Arabia; 2 Department of Chemistry, College of Science, Al-Zulfi Campus, Majmaah University, Al-Majmaah, Saudi Arabia; IIIT Kurnool: Indian Institute of Information Technology Design and Manufacturing Kurnool, INDIA

## Abstract

A novel membrane can enhance the efficiency of various industrial processes and help address critical issues. Membranes made of polyamide are widely used and successful in membrane separation processes. This paper outlines a viable method for creating a three-dimensional gyroid nanostructured polyamide membrane through selective laser sintering. This method has easy setup, fast membrane preparation, no pollution, and low preparation cost. It is better than old-style solvent casting methods, which have inadequate management over the membrane structure. The prepared membrane was characterised using various essential techniques, and its properties were examined. The size of the membrane was 3 × 3 cm. A negative skewness value of −0.107 and a surface roughness of 22.4 nm indicate the presence of small peaks and high roughness. The CHN analysis shows the presence of 8.310% nitrogen, 42.100% carbon, 4.327% hydrogen, and 19.076% sulfur in the prepared membrane. The compressive strength of the membrane was calculated to be approximately 30 Mpa. A preliminary experiment on oil-water separation was conducted to address the growing issue of industrial oily wastewater. This study highlights the critical impact of surface properties on enhancing membrane performance, further solidifying their significance in membrane technology. This study provides insights for optimising membrane performance in future research.

## Introduction

The first additive manufacturing (AM) system emerged in the 1980s when Dr. Hideo Kodama, an inventor, applied knowledge from 3D scanning and layering patterns to create a prototyping machine. Since then, AM has experienced a significant evolution. The development of stereolithography, selective laser sintering, 3D printing, laser additive manufacturing, fused deposition modelling, PolyJet printing, and rapid prototyping has led to significant advancements in this field. These ongoing improvements have resulted in new applications for additive manufacturing in different sectors and the creation of novel research areas. AM significantly reduces lead times compared to traditional methods. AM increases design freedom for engineers, allowing them to create parts that transcend the limitations of conventional manufacturing processes. Modern 3D printing platforms provide several benefits compared to earlier additive manufacturing technologies. These advantages include improved reliability, faster speeds, user-friendly interfaces, a wider range of material options, energy efficiency, enhanced safety, reduction of logistics costs, minimised risk in supply chain operations, cloud connectivity, sustainability, more environmentally friendly production methods, improved design, reduced transportation costs, and increased manufacturing speed. The future of AM is promising, with significant growth expected in the coming years. This growth will likely continue as more industries adopt 3D printing technology and the costs of 3D printers decline. New medical devices are constantly being developed using 3D printing technology. AM technology has had a significant impact on the aerospace and automotive sectors. Shortly, additive technology is anticipated to gain broader application across various industries. Combining natural fibers and synthetic fillers, hybrid composites have recently gained considerable attention [1,2].

Selective laser sintering (SLS) is a widely used AM technique involving thin layers of powdered polymeric materials spread across the printing bed platform using a laser as the power source. Since different materials absorb light at different rates, choosing the right laser for SLS depends heavily on the material being processed. SLS supports a wide range of 3D printing materials, but polyamide (also known as nylon)-12 (PA-12) is the most popular owing to its excellent mechanical properties, good chemical resistance, high dimensional stability, low moisture absorption, fuel resistance, impressive wear properties, and rough surface. By using a computer-aided design (CAD) model, the SLS creates 3D-shaped structures layer by layer within a short amount of time. SLS contributes to the synthesis of polymer nanocomposites in an innovative way. The SLS process is adept at manufacturing parts from various materials. SLS printing has been a widely favoured option for engineers and manufacturers for many years. SLS offers numerous advantages compared to traditional manufacturing processes, including lower cost per part, increased productivity, lesser heating, better finishing, rapid prototyping, custom manufacturing, superior mechanical performance, high reliability, and electrical and thermal conductivity. There are numerous applications and innovations of SLS across various sectors [3]. SLS manufactures parts from polymers, metals, and ceramics [4]. It is successfully used in manufacturing alumina-molybdenum nanocomposites [5], tungsten disulfide reinforced polyamide-12 nanocomposites [6], nano Al2O3-infused polyamide [7], polyamide/graphene nanocomposite [8], Polymer-Coated Molybdenum powder [9], etc. Recent advancements in SLS suggest its potential application in the preparation of membranes. A porous membrane was fabricated using SLS to separate oil from water and immiscible organic mixtures [10]. SLS prepared chitosan and thermoplastic polyurethane composite membranes for adsorption and catalysis [11]; polyamide-12 membranes were also produced through SLS for microfiltration [12].

The production of membranes is essential for water purification and energy consumption. Liquids or gases are separated by membranes, which are thin films. Polymer membranes are used in a wide range of applications. 3D printing has the potential to transform traditional membrane applications in several ways. It allows for creating membranes with complex geometries and tailored properties that can be customised for specific applications, which is difficult to achieve with traditional manufacturing techniques. Different materials can be used in 3D printing to enhance membranes’ performance, permeability, selectivity, and durability. 3D printing can seamlessly integrate membranes into larger systems or structures, improving efficiency and functionality. Designing and manufacturing more efficient membranes can contribute to energy savings and reduce environmental impact, supporting sustainable practices. Developing new membranes through 3D printing occupies an essential portion of ongoing research.

Thoroughly characterising membranes is crucial for understanding and optimising their performance. It helps understand how they function, their strengths, and areas where they can be modified. This deep dive into their characteristics ensures they are practical and reliable. Polyamide was selected to make membranes durable based on its thermal, chemical, and mechanical properties. A gyroid nanostructure was designed to ensure the membrane has a narrow pore size distribution, high porosity, and high selectivity. This study explores the surface, functional, thermal, and mechanical properties of SLS made 3D shaped gyroid cubic lattice structured polyamide nanoporous membrane (GPM = Gyroid Polyamide Membrane) and comprehensively studied by SEM, EDX, AFM, TEM, FTIR, and TGA. Its findings will serve as the basis for future membrane research.

## Materials and methods

### Overview of gyroid structure

Lattice structures contain macroscale pores, and macroscopic properties exhibit outstanding mechanical properties in various engineering applications. Gyroid lattice structures are complex 3D geometric patterns comprising triple periodic minimal surfaces (TPMS) with zero mean curvature and minimised local area. The structure has the smallest surface area yet fits within defined boundaries. In this space, two parallel regions are intertwined by smooth, continuous surfaces. The microstructure of butterfly wings and bird feathers inspires gyroid lattice structures. Alan Schoen introduced the concept of the gyroid structure in the 1970s, showcasing the Blue Morpho butterfly as a classic example found in nature [13]. With the advancement of additive manufacturing (several 3D printing technologies), the gyroid structure has become an exciting and distinctive infill pattern for developing new variations of existing materials. Standard methods to manufacture Gyroid lattice structures using additive manufacturing (AM) techniques are Laser Powder Bed Fusion, Fused Filament Fabrication, PolyJet Printing, and SLS. These methods allow for the precise and efficient creation of gyroid lattices, valued for their high strength-to-weight ratio and energy absorption capabilities. SLS provides an effective method for fabricating such complex structures. A laser that sinters powdered material, typically nylon or other polymers, layer by layer, was chosen. Commonly used materials are nylon, polyamide, and other thermoplastics. The benefits of using SLS include strong mechanical properties and the fact that there is no requirement for support structures.

Smaller unit cell sizes in gyroid lattice structures exhibited better performance and increased relative densities [14]. In a comparative study, the Gyroid structure with a shell design demonstrates superior energy absorption efficiency, particularly at lower relative densities. A Gyroid lattice made of polyamide (PA)-12 with a relative density of 0.23 exhibited a 68% energy absorption efficiency [15]. The latest study’s results indicate that the increased specific surface area of the Gyroid lattice structure enhances powder adhesion [16]. Gyroid structures with a density of 40% demonstrate remarkable compressive strength, closely mimicking the biomechanical properties of bone. A significantly increase the material’s elastic modulus and energy absorption can be achieved by adding 8% tantalum (Ta), making it more suitable for dynamic load-bearing implants. This improvement ensures better performance and longevity under varying loads [17]. The gyroid structure yields superior elongation and impact strength results compared to solid structures [18].

### Materials and instruments

Polyamide 12 (PA-12) + carbon fresh and smooth powder with excellent surface resolution was purchased from Sinterit, Poland. The PA-12 smooth has a refresh rate of 22%, which allows it to be used as 78% recycled powder. The refresh rate is the ratio of the minimum amount of fresh powder needed in a mixture of materials used in an SLS 3D printer. The material will be more cost-efficient if the refresh ratio is lower. PA-12 smooth is a cost-efficient rigid polyamide. The colour of the powder is navy grey. The mechanical properties of the powder are: Tensile Strength = 32 MPa, Tensile modulus (Young) = 1470 MPa, Flexural strength = 47 MPa, and Impact strength as per unnotched Charpy method = 16 kJ/m^2^. The thermal properties of the powder are: Melting point = 185°C and Heat deflection temperature = 68 °C.

Selective Laser Sintering (SLS): The LISA Pro 3D printer was purchased from Sinterit Company in Poland. This model features an open printing system, a larger build volume, excellent print quality, a wide range of applications, and a reputation for reliability. It is user-friendly and dependable, much like its predecessor. The design is ultra-durable, with a 110 × 160 × 250 mm build area, and includes an integrated nitrogen chamber for improved material compatibility. The Lisa Pro has a maximum diagonal print volume of 301 mm (11.8 inches) for polyamide materials and 313 mm (12.3 inches) for flexible materials. It uses an IR Laser Diode with a power of 5 W at a wavelength of 808 nm. The printer’s maximum operating temperature is 392 °F (200 °C).

### Membrane fabrication using 3D model and design printing

**Fig 1:**
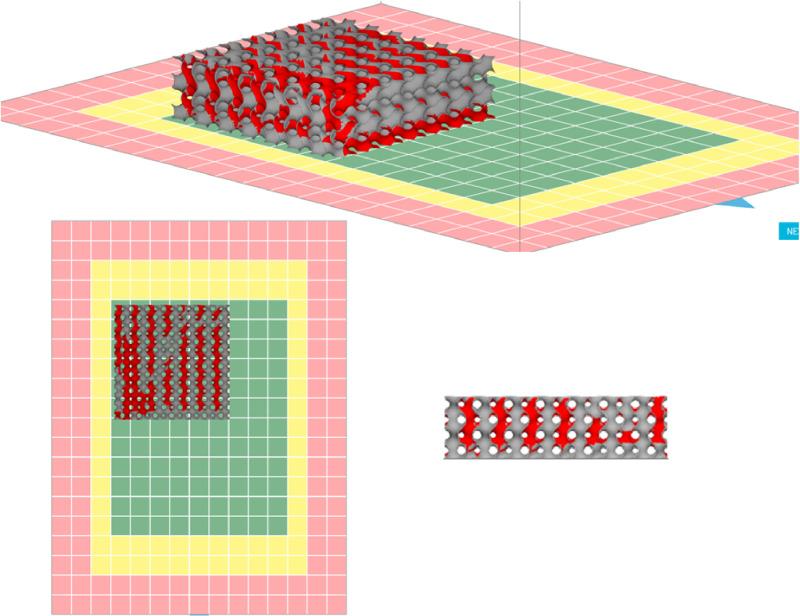
Membrane design by Sinterit studio.

**Fig 2:**
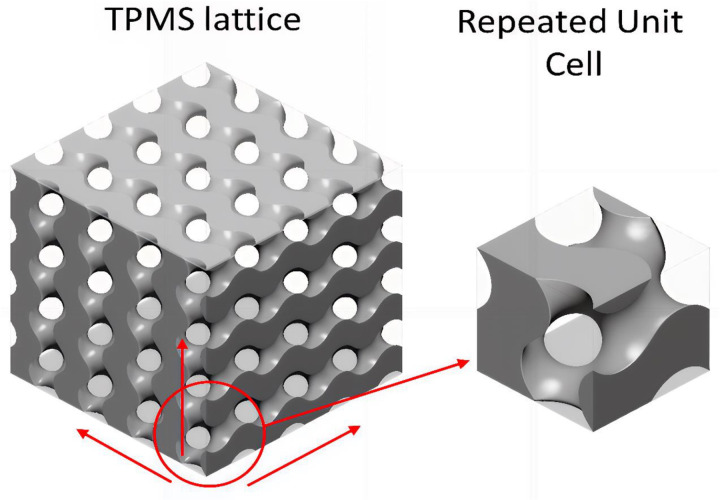
Design of Gyroid structure [19].

**Fig 3:**
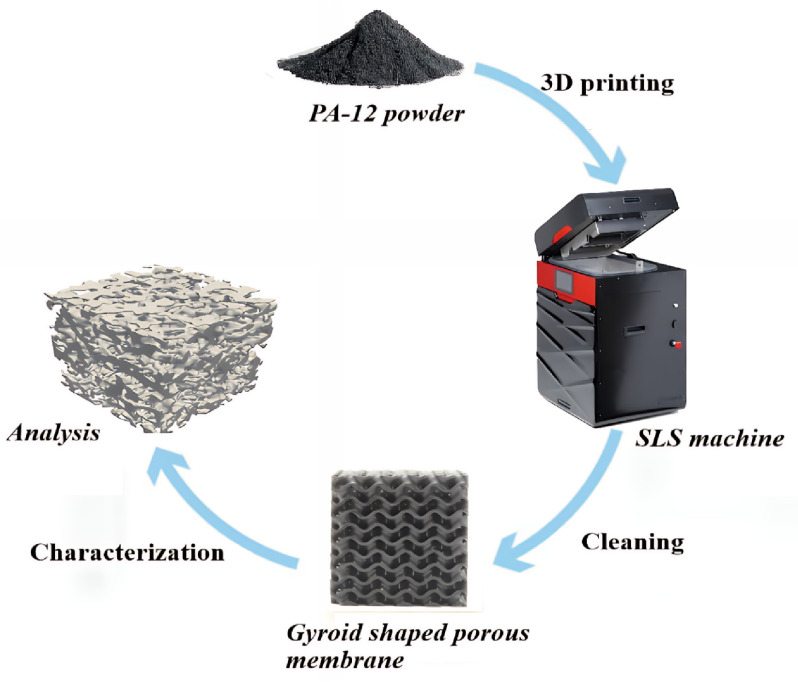
Visual representation of the fabrication process.

The gyroid design was scheduled to be 3D printed. It was initially designed using the GrabCad platform, exported in STL format to the software Sinterit studio, and then 3D printed using the SCode file SM 4. code (Fig 1). The geometry of a gyroid is similar to the structure of crystals, as it comprises a multidirectional, complex, three-dimensional set of minima (Fig 2) [19]. The size of the membrane was 3 × 3 cm. The material used to print the membrane was PA-12 smooth. Printing time, nitrogen consumption, plate temperature, warming layers, layer thickness, and waiting time after each layer were 16 h, 0.48 m^3^/h, 175 °C, 133 layers, 1 mm, and 10 s, respectively. After printing, the structures were carefully cleaned using a Sinterit sandblaster and soft brush to remove unsintered sticking powder. In this study, the PA-12 powder mixture was kept via the SLS technique for the 3D printing process. Fig 3 shows the synthesis process. This preparation method presents an uncomplicated, easy, and practicable way to produce 3D membranes with specific properties that can be used in many industrial applications.

### Membrane characterisation

A Thermo Scientific™ Nicolet™ iS20 Fourier Transform Infrared Spectrometer (Waltham, MA, USA) analysed intermolecular compatibilities and interactions between components. The samples were scanned in triplicate over the 4000–550 cm^−1^ range, with the spectral resolution set at 4 cm^−1^. An average of 32 scans per sample was recorded. Scanning electron microscopy, atomic force microscopy, and CHN elemental analysis were conducted using the JEOL JSM-7100F (Tokyo, Japan) field emission electron microscope at a resolution of 1.2 nm. Thermogravimetric analysis was performed using TA Instruments SDT-Q600 (USA) from 25.00°C to 700.00°C at 10.00°C/min.

### Mechanical testing

The polyamide specimen was subjected to a compression test to determine its mechanical properties. The compression test was conducted using a guntHAMBURG WP 300 material test machine. The temperature remained constant during the experiment.

### Oil/Water separation

Water treatment eliminates pollutants and dangerous constituents from the water samples [20,21]. Polyamide and membranes are both established and highly sought-after approaches for water treatment [22–25]. A preliminary oil/water separation experiment was conducted to have an initial look at the possible industrial application of the prepared GPM. The oil-water mixture was created by combining water and sesame oil in a weight ratio of 70:10 v/v. The prepared GPM membrane was secured between two glass containers measuring 30 mm in diameter. The prepared oil-water mixture was dispensed into the membrane, and the separation was achieved solely through gravity.

## Results and discussion

### Characterisation

#### SEM.

**Fig 4:**
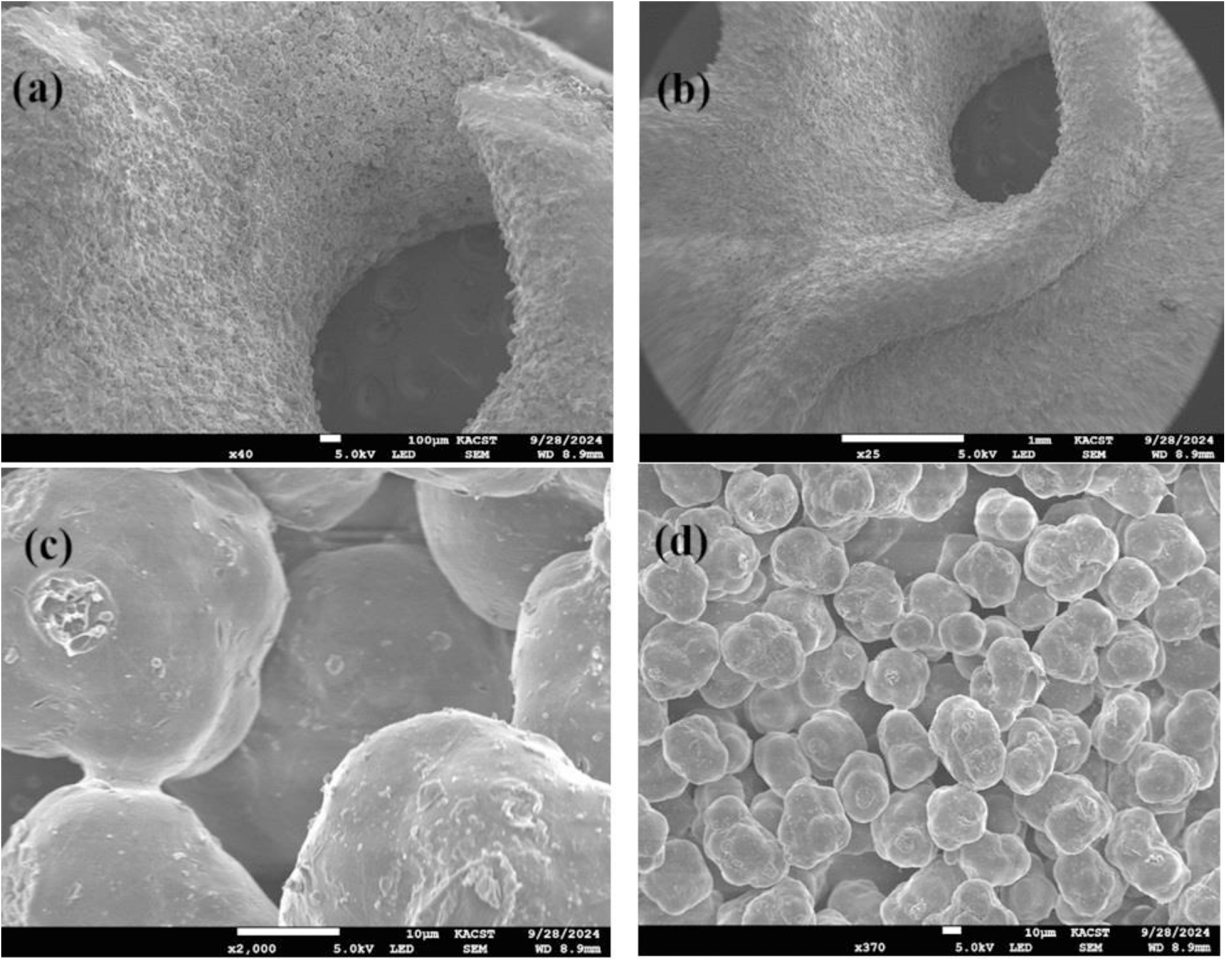
SEM analysis of GPM.

An SEM analysis provides valuable insight into membrane surface structure and cross-sectional design. Figures (Fig 4a and b) show that the GPM membrane exhibited a typical polyamide structure with dense spheres. SEM micrography of GPM shows the wide membrane surface area. The SEM image of the GPM shows that it has a smooth surface and low roughness. This soft and packed morphology is due to the binding effect of PA. The morphology is spongy, interconnected, and has a porous structure. A thin, porous layer around the membrane walls adhering to the surface of the membrane with abundant tiny pores is also visible. The porous structure of GPM provides a larger surface area, and more sites give a spherical shape. The agglomeration of the pores covers all the membrane surfaces, and they are uniformly dispersed on the surface, enhancing the performance of the polyamide material via a micro-aggregate effect. High magnification shows cracks in the pores due to the high porosity and the large number of inclusions within the part resulting from unfused powder particles (Fig 4c and d). Such structure is desirable as it allows greater penetration of contaminated water and, hence, more significant adsorption of target species.

The internal geometry of the gyroid pattern and particle surfaces is different. Gyroid holes have a rough surface and ridge-and-valley structure typical of polyamide layers. In contrast, the surface of the PA-12 is smooth and has a modular structure that is closely packed. According to Villalobos et al., the polyamide active layer of a SW30XLE RO membrane has different surfaces on the front and back [26].

#### AFM.

**Fig 5:**
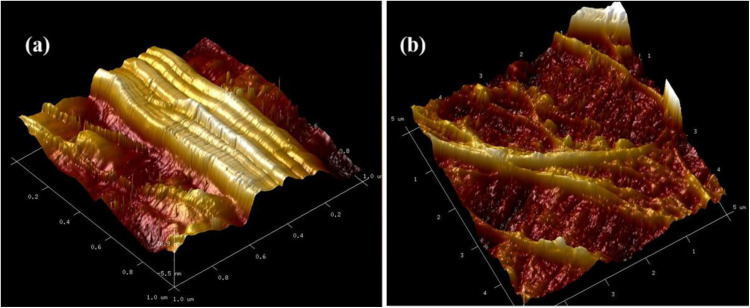
AFM analysis of GPM.

AFM technique is beneficial for analysing membrane properties, such as roughness, pore size, mechanical properties, and hydrophilicity. In the AFM image of GPM, the ridge-and-valley structure was observed (Fig 5a and b). The surface is rough with a thick skin layer, and big bumps occur on the membrane [27]. A continuous surface with ups and downs on the membranes, like a rising and falling hill structure, can be observed. The tip moves up and down over a wide range when the surface includes pores and peaks. This large number of small spikes, peaks, and valleys show good compatibility of PA-12 within the gyroid matrix. This led to a suitable surface structure being developed in the membrane. A negative skewness value of −0.107 indicates the presence of small peaks with deep and narrow valleys. The surface roughness (Ra) of GPM was measured as 22.4 nm, showing a high mean roughness of the membrane. The higher surface roughness of the GPM membrane could be related to the possible formation of PA-12 agglomerates at high concentrations in the gyroid matrix, which brings more peaks to the membrane surface [28].

#### CHN analysis.

CHN analysis is a practical elemental analysis measured using gas chromatography to detect the sample’s carbon, hydrogen, and nitrogen elemental content. The results showed the presence of 8.31% nitrogen, 42.10% carbon, 4.327% hydrogen, and 19.076% sulfur in GPM. It has a value of 5.0301 C/N ratio and 9.7296 C/H ratio. Polyamide is a repeated amide bond, has 12 carbon, and its chemical formula is [(CH_2_)_11_C(O)NH]_n_. The CHN analysis results confirm all the molecules in the gyroid structure. H_2_S is a gas that passes through the membrane barrier layer and, under certain conditions, precipitates as elemental sulfur in the membrane micropores.

#### FTIR.

**Fig 6:**
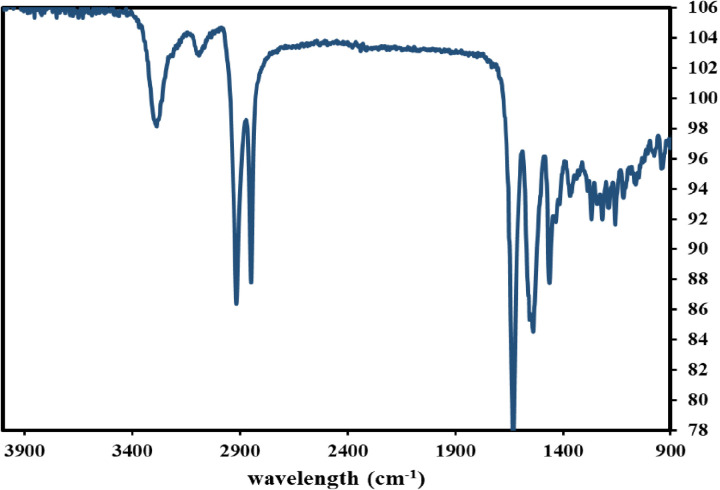
FTIR analysis of GPM.

FTIR spectroscopy was used to understand GPM’s chemical nature and structure (Fig 6). The FTIR spectra show that PA-12 exhibits a broad peak at 3286.59 cm^−1^, attributed to hydrogen-bonded N–H stretching vibration. The stretching peak for N-H is similar to that for O-H [29]. The hydrogen bond strength of N-H bonds is weaker than that of O-H bonds, which is why N-H stretches are narrower than O-H stretches [30]. The peaks at 2917, 2865, and 2847 cm^−1^ are assigned to asymmetric C-H stretching vibration in methyl groups of side chains [31], significantly influencing the aggregated structure of polymers [32]. The peak at 1680 cm^−1^ corresponds to conjugated C = O stretching peaks in all amides [33]. The peak at 1585 cm^−1^, associated with the C-C bond in aromatic compounds, is typical of the sulfone group [34]. The peak at 1537 cm^−1^ is attributed to the amide-II band and CH_2_ asymmetric deformation [35]. An additional band was observed at 1482 cm^−1^, attributed to the C-N stretching vibration. The peak of 1463 cm^−1^ is ascribed to the bending deformation of the methylene CH_2_ group.

#### TEM.

**Fig 7:**
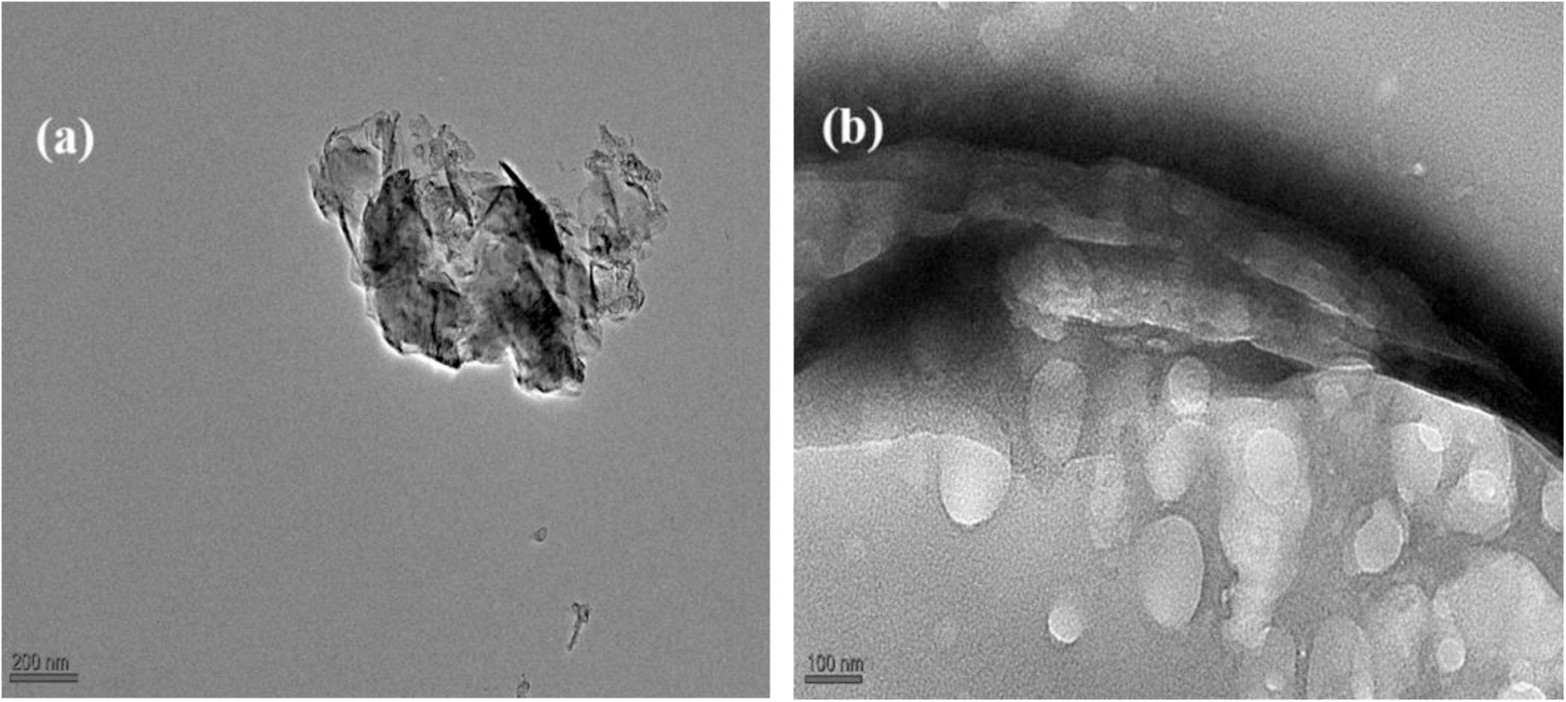
TEM analysis of GPM.

TEM is utilised to analyse the pore morphology and structure of the membrane at an atomic scale. Fig 7 shows agglomerates of poorly crystalline PA-12 particles were noted. PA-12 particles are spherical, round, and polydisperse and can easily be seen as a bubble-like structure. Polyamide bulges are common and randomly distributed over the membrane surface. The majority of the polyamide particles are homogenous and uniform in thickness. Careful observation suggests the gyroid membrane has a dark, dense core base layer and round PA-12 particles extended outwards.

#### TGA.

**Fig 8:**
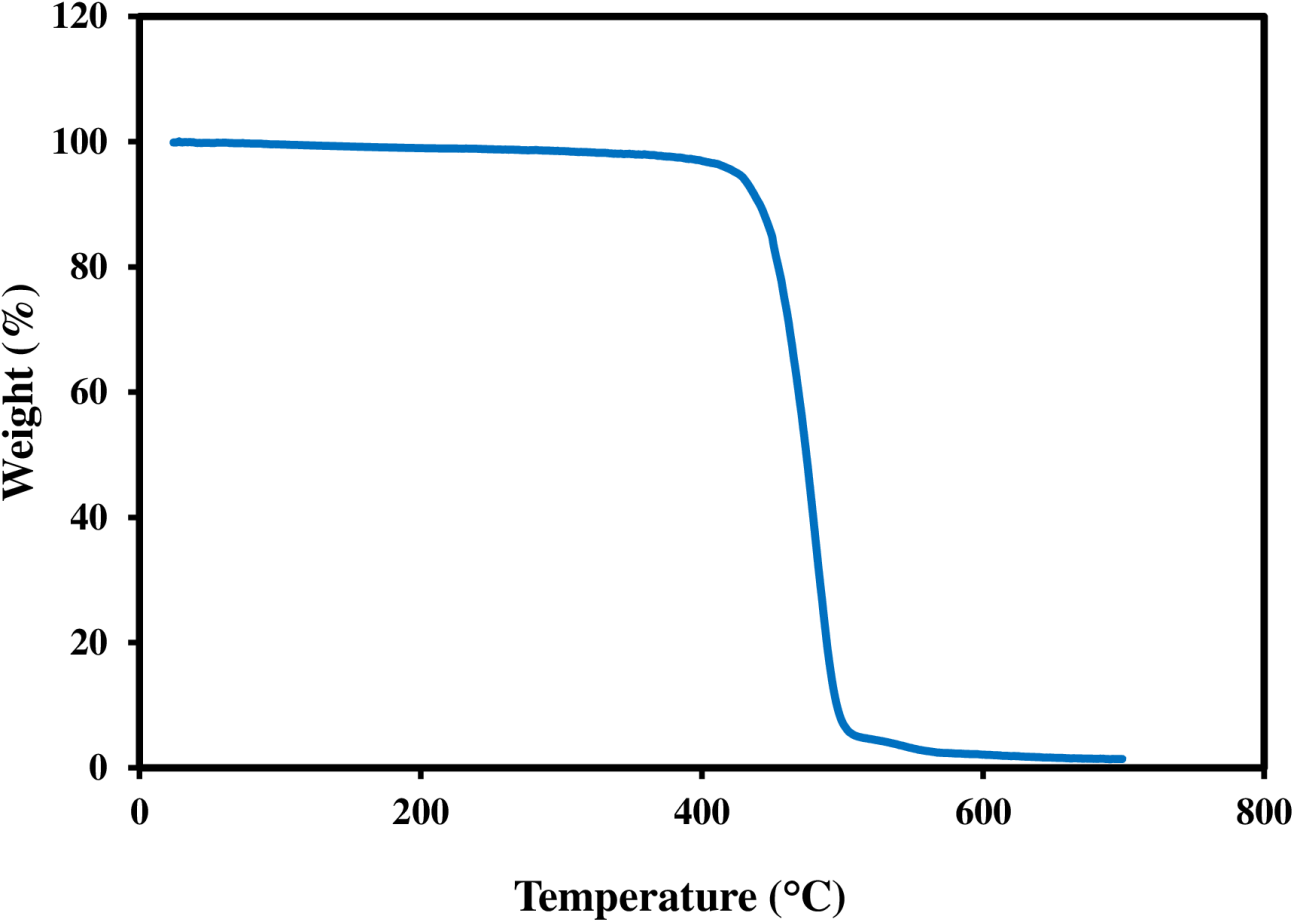
TGA analysis of GPM.

TGA is mainly used to assess the thermal stability of polymer membranes. Fig 8 shows the high thermal stability of GPM; only a minor weight loss was observed from 100 to 425 °C. The evaporation of the remaining water is what triggered this remarkable phenomenon. After that, significant weight loss is observed up to 500° C and approximately 98% of degradation is noted till to the completion of a reaction. As temperatures rise, the delicate balance of volatile substances shifts, leading to weight loss. The final stage of degradation involves the continued decomposition of polyamide (600–700 °C).

#### Mechanical strength.

**Fig 9:**
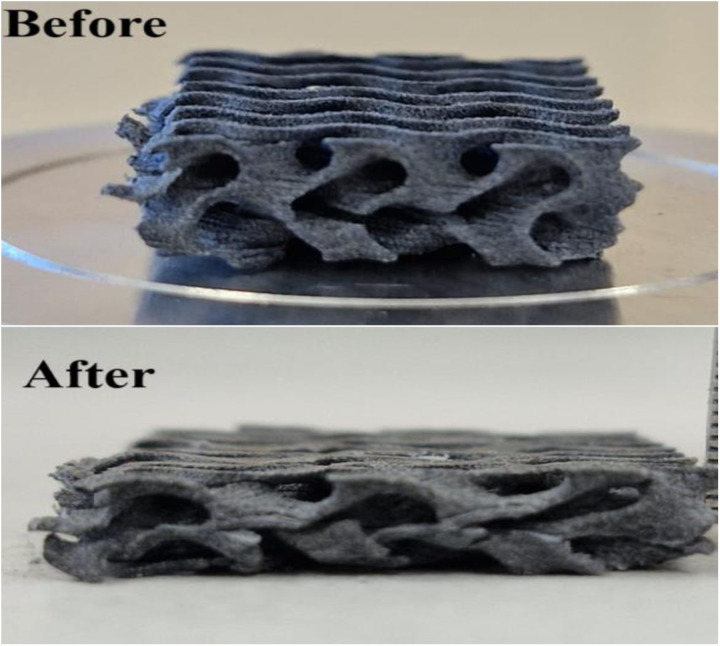
Effect of compressive strength on GPM.

The compressive strength of GPM was calculated to be approximately 30 Mpa, indicating that the prepared nanoporous membrane possesses excellent stability. The sintered part was not degraded, and the investigated range of laser power had no adverse impact on its compressive performance. Fig 9 shows that GPM absorbed a good amount of stress-strain, and there is no significant change in its lattice surface curvature. This enhances the material’s ability to resist deformation more efficiently. This outcome benefits structural applications to ensure excellent industrial use before rupture. It has been established that GPM is appropriate for application areas with a high energy absorption capacity. The Gyroid structure absorbs a significant amount of energy and exhibits excellent stability in energy absorption during compression. Laskowska et al. identified that all previously studied cylindrical Gyroid TPMS structures are predominantly governed by this deformation mechanism, which allows for greater design flexibility [36]. The compressive strength of PA-12/ Cement Composite at three laser power (watt) was 39 Mpa [37].

#### Oil/Water separation experiment.

**Fig 10:**
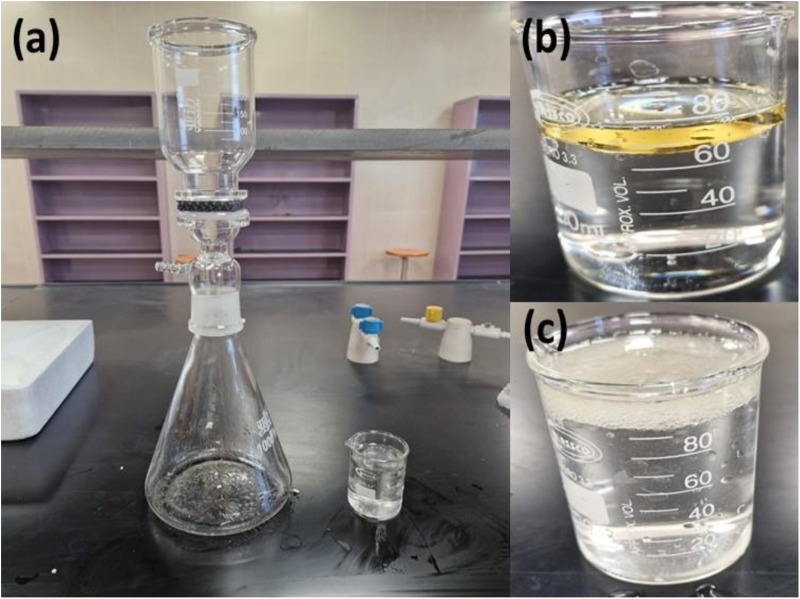
(a) Gravity filtration setup (b) before separation (c) after separation process.

The prepared GPM membranes, which have high porosity, show efficient separation for oil-water mixtures. The gravity-driven oil–water separation experiment was carried out using the setup shown in Fig 10a. The GPM membrane was positioned in the center of the device, and 80 mL of an oil-water mixture (with a volume ratio of 70:30) was poured into the glass tube. No external force drove the separation process. The water collected in the conical flask after passing through the membrane, whereas the oil remained above. Fig 10b and 10c show the images of the oil-water mixture before and after the separation, respectively. High separation efficiency is noted, and an apparent reduction in oil density was observed. This suggests that the prepared GPM is an effective candidate for treating industrial oil-polluted water and managing oil spill cleanup. However, oil droplets and emulsions were present in the separated water. This preliminary experiment was the first step in physically observing GPM’s work capacity in oil/water separation. To better examine the separation capability of the prepared GPM, an upcoming study aims to conduct a comprehensive investigation with an advanced experimental setup. This will involve measuring water contact angles and assessing chemical oxygen demand (COD) using oil samples such as gasoline, diesel, vegetable oil, hexane, and petroleum ether.

## Future perspective and conclusion

This study’s results confirm that the prepared membrane’s microstructure and properties are mechanically and chemically durable. The promising results show enhanced mechanical stability as GPM exhibits high strength and toughness. Mechanical properties are linked to membrane pore structure. The prepared nanoporous GPM showed good membrane properties and mechanical stability, which can be helpful in critical applications such as water treatment and oil-water separation. When an oil/water mixture was poured onto the membrane, water could pass through the membrane by gravity while oil was kept on the upper side. High separation efficiency was noted, and oil density was reduced. This indicates that the prepared GPM could be a strong candidate for industrial wastewater treatment. The chemical functionality of the GPM was confirmed by the characterisation techniques. Moreover, the facile preparation of GPM has a potential for practical applications in emulsified wastewater remediation. 3D membranes show increased filtration efficiency and notable developments in mechanical strength [38]. Gyroid structures combined with PA-12, as present in this study, exhibit the potential for creating strong composite materials suitable for use in diverse industries like aerospace and automotive, medical and water treatment. An area for future work could involve comparing different topologies of 3D-printed cores to compare and optimise their mechanical properties. This study set the stage for future work to optimise the membrane performance by testing various mechanical analyses, such as tensile strength and detailed oil/water separation. This method can also be applied to a broader range of polymers beyond polyamide-12, extending from lab production to small-scale manufacturing.
